# Comprehensive review of hybrid aortic arch repair with focus on zone 0 TEVAR and our institutional experience

**DOI:** 10.3389/fcvm.2022.991824

**Published:** 2022-09-15

**Authors:** Saket Singh, Stevan S. Pupovac, Roland Assi, Prashanth Vallabhajosyula

**Affiliations:** Section of Cardiac Surgery, Department of Surgery, Yale University School of Medicine, New Haven, CT, United States

**Keywords:** aortic surgery, hybrid aortic arch repair, stent-graft, TEVAR, thoracic endovascular aortic repair, aortic pathology

## Abstract

Even with increasing operator experience and a better understanding of the disease and the operation, intervention for aortic arch pathologies continues to struggle with relatively higher mortality, reintervention, and neurologic complications. The hybrid aortic arch repair was introduced to simplify the procedure and improve the outcome. With recent industry-driven advances, hybrid repairs are not only offered to poor surgical candidates but have become mainstream. This review discusses the evolution of hybrid repair, terminology pertinent to this technique, and results. In addition, we aim to provide a pervasive review of hybrid aortic arch repairs with reference to relevant literature for a detailed understanding. We have also discussed our institutional experience with hybrid repairs.

## Evolution of hybrid aortic arch repair

### Aortic arch surgery

First report of successful open aortic arch surgery was by Cooley in 1951, when he performed tangential excision and lateral aortorrhaphy for a saccular aneurysm of the aortic arch (1). Cooley in 1955 then reported total excision of the aortic arch aneurysm in a 49-year-old-man using a custom made Ivalon prosthesis. He used temporary shunts, made of Ivalon, to maintain cerebral perfusion as cardiopulmonary bypass machine was not available to Houston group at the time (2). First description of replacement of the aortic arch using a pump oxygenator was also by the DeBakey group in 1956 (3). They employed antegrade perfusion of the innominate and left carotid artery for the procedure and homograft was used as a prosthesis. Randall Griepp, in his seminal paper in 1975, introduced the concept of hypothermic circulatory arrest in aortic surgery, where he reported four cases of aortic arch replacement, with one mortality (4).

### Open aortic arch surgery: Current status

Open aortic arch surgery is increasingly more common with a little improvement in mortality and neurological complications over decades, which have been an Achilles' heel in the progress of aortic arch surgery (5, 6). A review of institutional databases and registries has revealed that 7–28% of patients needing ascending aortic and arch surgery for Stanford type A aortic dissection are deemed inoperable due to their comorbidities and other high-risk features like old age, fragility, and previous cardiac surgery. We also know that only 34% of these patients have truly prohibitive risk factors like advanced dementia and late-stage malignancy. These high-risk patients undergoing conservative management have extremely high in-hospital mortality of 58–66% (7, 8).

The apprehension of performing aortic arch repair in a frail patient, or in a redo patient is not without merit, as shown in a retrospective review of a prospective single-institution database with a significant increase in 30-day mortality (8.8 vs. 1.4%) for frail patients (9). Rylski et al. have shown that redo aortic surgery cohort are significantly older and five times more likely to have underlying coronary artery disease., with resultant higher in-hospital mortality (25 vs. 12%, *p* < 0.01) and significantly lower one and ten-year survival (10).

### Endovascular approach to the aorta

The initial description of the feasibility of stent-graft combination for endovascular aortic deployment was from the JC Parodi group from Argentina in 1991, when they reported the outcome of animal experiments and five patients with abdominal aortic aneurysm (11). Professor Nicolai Volodos, from the former USSR, first performed endovascular exclusion of descending thoracic aortic (DTA) aneurysm (12). Volodos et al. are also credited for the first hybrid aortic arch repair. The Patient had a distal aortic arch aneurysm, which required debranching of the supra-aortic vessels and antegrade deployment of a stent-graft (13). The patient survived for over 24 years without any reported complications (14).

The feasibility of Thoracic Endovascular Aortic Repair (TEVAR) was established by Dake et al. in their report of thirteen patients with DTA aneurysms and dissections (15). They reported successful endovascular deployment of a dacron graft mounted on a self-expanding stainless steel frame in all the patients, with no death or neurological complications. In addition, twelve out of thirteen patients demonstrated evidence of positive aortic remodeling during an average follow-up of 11.6 months. First FDA approval for the use of a stent-graft for the treatment of DTA aneurysm was granted in March 2005 for the GORE TAG device (GORE TAG thoracic nitinol endograft; WL Gore & Associates, Inc, Flagstaff, Ariz).

### Hybrid aortic arch repair

The first account of staged hybrid aortic arch repair (HAR) was from Czerny et al. in 2002 in an 80 year-old-male with a contained rupture of the aortic arch involving the origin of the left common carotid artery (LCA) (16). Feasibility and safety of HAR in high-risk patients was established by the early work of Saleh et al. when they reported technical success in all fifteen patients with aortic arch aneurysms (17). All the stent-grafts and bypassed supra-aortic vessels were patent during a mean follow-up of 18 ± 2.5 months, with no evidence of endoleak, graft migration, or neurologic complications. Weigang et al. also reported their experience with aortic arch debranching followed by stent-graft deployment in the native ascending aorta in a cohort of 26 high-risk patients. They had technical success in all the patients, and there was no incidence of stroke or paraplegia (18).

Demonstration of the feasibility and safety of HAR in high-risk patients with aortic pathology with its ability to avoid hypothermic circulatory arrest (HCA) attracted the attention of our surgical community and industry, leading to rapid advances in the hybrid technology.

## Nomenclature and classifications

### Landing zones

**Figure 1:**
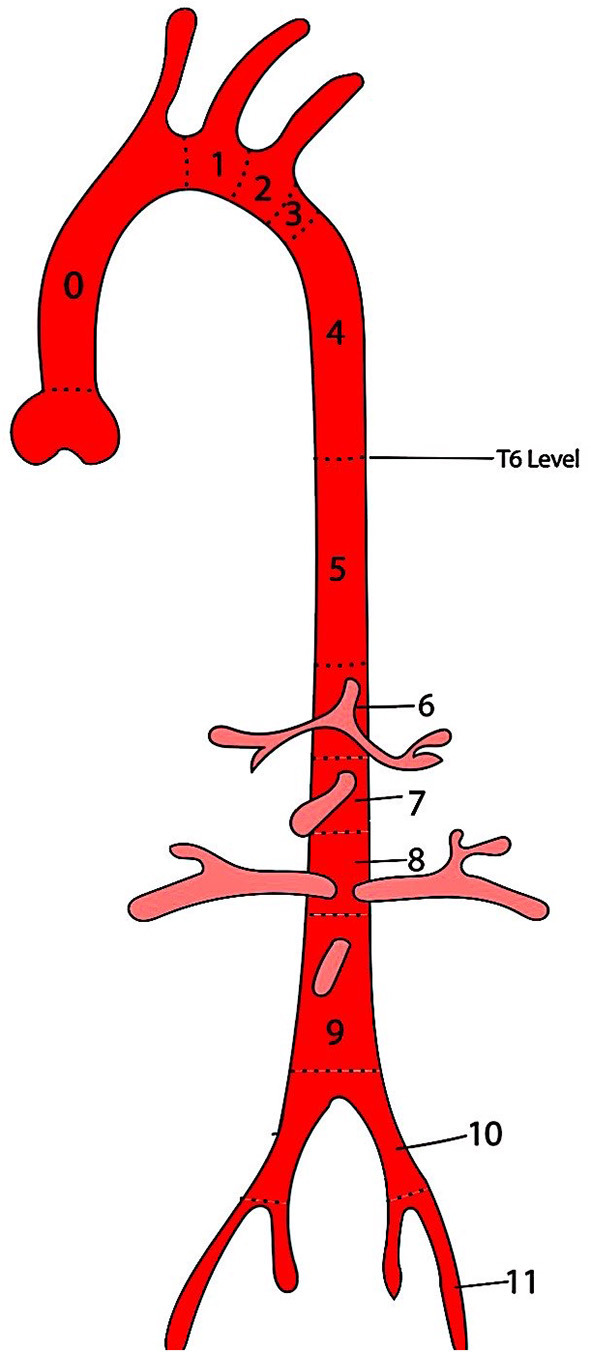
Ishimaru anatomical landing zone map. The positions of the proximal and distal ends of the stent-graft are described by zones (Z), which are based on lines drawn from the distal side of the branch arteries from the aorta.

The first attempt at anatomical classification for endografting was by Balm et al. They suggested a system depicting the location of a stent-graft deployment by a line drawn from the distal side of each branch artery ostium on the aortic arch, thus providing information about the arteries covered by the stent-graft (19). Ishimaru presented his “anatomical endograft zone map” during the first International Summit on Thoracic Aortic Endografting held in Tokyo in 2001 (20). He proposed a “zone system” to define the proximal landing site with the intent to streamline the comparisons of different indications and outcomes across the institutions. This classification system was adopted across the globe, which led to the expansion a year later to include a distal landing zone (Figure 1) (21). This system requires clinicians to mention the nature of the stent-graft at the landing zone, i.e., covered vs. uncovered, and the graft material.

### Classifications

**Figure 2:**
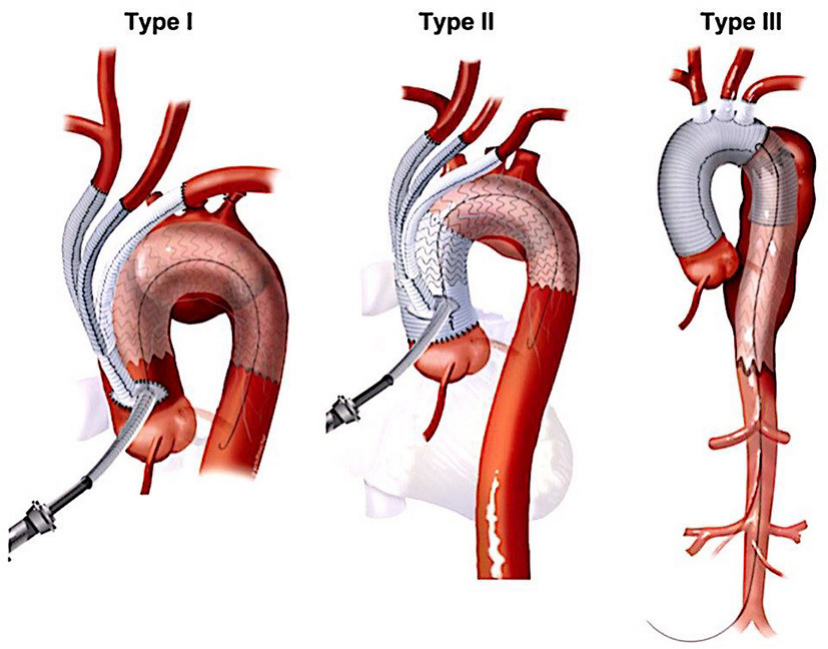
Major types of hybrid arch repair (22). HAR, hybrid arch repair.

Numerous variations in techniques and approaches employed for HAR stemmed from the complexity of the procedure, given the shape of the aortic arch and crowding of supra-aortic vessels in a tight space. The endovascular component of the HAR is based on the exclusion principle that does not require replacement of the diseased segment of the aorta. HAR entails debranching of supra-aortic branches to create a landing zone where it does not exist for simultaneous or staged placement of the stent-graft to exclude the diseased aortic segment. Bavaria et al., from the University of Pennsylvania, proposed the universally accepted and most straightforward classification for the HAR (23). They described three types of hybrid procedures (Figure 2, Table 1). Type I HAR is employed for disease limited to the aortic arch with a healthy ascending aorta suitable for deployment of a stent-graft in zone 0. This entails debranching and re-implantation of supra-aortic vessels on the proximal ascending aorta to provide long enough segment for the proximal landing zone (PLZ). Type I HAR is sub-divided into Type Ia, if debranching was done without cardiopulmonary bypass (CPB), and Type Ib, if CPB was required for an end-side anastomosis of a multi-branched graft used for debranching on the proximal ascending aorta. Type II HAR is when the ascending aorta is unsuitable for endograft deployment and is replaced with a dacron graft to create a PLZ for a stent-graft after debranching the arch. Type II hybrid procedures can involve a brief period of circulatory arrest to accomplish a hemiarch replacement or a zone 1 or 2 distal anastomoses as dictated by the extent of the disease. Type III HAR is required when descending aorta is not ideal for stent-graft deployment. Type III repair, commonly used for patients with diffuse aortic disease or “mega-aorta” syndrome, requires open replacement of the arch followed by deployment of the elephant trunk in the proximal descending aorta, which will then serve as the proximal landing zone for an interval TEVAR.

**Table 1:** Different types and subtypes of Hybrid aortic arch repair based on the extent of endovascular coverage and open component.

**HAR type** **(23, 24)**	**Subtype**	**Indication based on the** **disease extent**	**Open component**	**Endovascular** **component**	**Comments**
Type I		Disease limited to the aortic arch	Debranching with anastomosis to the proximal ascending aorta	Stent-graft deployment in zone 0	
	Type Ia		Type I debranching without CPB		
	Type Ib		Type I debranching under CPB		
	Type In		Stent-graft deployed in native ascending aorta		Risk of RTAD
	Type Id		Stent-graft deployed in the Dacron graft in zone 0		
Type II		Disease involving the ascending aorta	Ascending aorta replacement to create a landing zone with debranching to the Dacron-graft	Stent-graft deployed in the Dacron-graft	Under CPB. Brief period of circulatory arrest required for hemiarch, zone 1 or zone II anastomosis
Type III		Disease involving the descending aorta/diffuse aortic involvement	Replacement of the aortic arch	Elephant trunk in the proximal descending aorta	
	Type IIIc	Conventional elephant trunk			
	Type IIIf	Frozen elephant trunk in the descending aorta			
Zone 1		Disease limited to the aortic arch	Cervical approach for Supra-aortic vessel transposition/bypass	Stent-graft deployed in Zone1	Bilateral cervical approach for debranching
Zone 2		Disease limited to the distal arch	Cervical approach for Supra-aortic vessel transposition/bypass	Stent-graft deployed in ZoneII	Unilateral cervical incision for left carotid to subclavian bypass/transposition
Zone 0		Landing zone not available in the aortic arch	Cervical debranching as in Zone 1	Stent-graft deployed in Zone 0	Cervical debranching similar to zone 1 HAR, with stent-graft deployment in Zone 0
	Zone 0s		Snorkel stent-graft to perfuse innominate artery		
	Zone 0b		Single branched stent-graft to perfuse innominate artery		

Table also provides a brief specifics of the procedure in the comment column. CPB, cardiopulmonary bypass; HAR, hybrid arch repair; RTAD, retrograde type A aortic dissection.

Based on the advances in the field of aortic arch surgery, Hughes et al. expanded upon the previous classification by sorting HAR into “zones” and “types” (Table 1) (24). As per this classification a hybrid repair is categorized as “type x” if it involves sternotomy. Hybrid repair involving cervical approaches for supra-aortic debranching ares categorized as “zone x.” Type I HAR is further categorized as type In when the stent-graft is deployed in the native ascending aorta and type Id if the PLZ is in the previously replaced ascending aorta Dacron graft. Type Id hybrid procedures are mostly done in patients with prior Stanford type A dissection repair. Type III HAR group was broadened to include a type IIIf variant when a frozen elephant trunk was combined with a total arch replacement. Type IIIc HAR is when repair involves arch replacement with a conventional elephant trunk.

Zone 0 HAR involves debranching the supra-aortic arteries through a cervical incision and deployment of endograft in zone 0. Zone 0 is classified as zone 0s when a snorkel or parallel endograft is deployed in the innominate artery to maintain flow in the debranched supra-aortic arteries. Zone 0b HAR points to a branched aortic endograft to perfuse the innominate artery. Zone 1 HAR means cervical debranching followed by deployment of the stent-graft in zone 1.

Bavaria group also proposed a classification system for TEVAR based on the extent of coverage of the DTA (Table 2). Type A TEVAR is stent-graft coverage from LSA to the 6th thoracic vertebrae (T6), type B is from T6 to the celiac axis, and type C when the endograft extends from LSCA to the celiac axis (25).

**Table 2:** Classification of TEVAR based on the extent of coverage of the descending thoracic aorta.

**TEVAR type** **(25)**	**Extent of endovascular coverage of the DTA**
**Type A**	LSCA to the level of T6
**Type B**	T6 level to the celiac axis
**Type C**	LSCA to the celiac axis

DTA, Descending Thoracic Aorta; TEVAR, Thoracic Endovascular Aortic Repair.

## Different approaches

### Type I HAR

Type In HAR is a safer approach when there is >2 cm of the healthy native aorta or at least 4 cm of Dacron-graft in zone 0. Endograft can be deployed antegrade through a dedicated limb of the multi-branched graft used for debranching or in a retrograde fashion using the ileo-femoral approach. Retrograde deployment can be staged or in the same setting.

Ganapathi et al., reported a striking decrease in the incidence of type I endoleak with an increase in the length of PLZ in the Dacron graft from <2.5 cm to 4–5 cm (13.3 vs. 0%, *P* = 0.03) (26). Retrograde type A aortic dissection (RTAD) is a major limitation of type In HAR, especially if the diameter of native ascending aorta is more than 4 cm. Anderson et al. in a retrospective study reported 11.1% incidence of early RTAD in a group of 27 patients undergoing type In HAR. Increased rate of RTAD translated into significantly higher 30-day mortality in type In HAR compared with type Id HAR (29.6 vs. 9.5%) (27). Nevertheless, type I HAR remains a promising approach for high-risk patients, given the ability to avoid CPB and circulatory arrest, especially in the setting of prior type A aortic dissection repair.

### Type II HAR

Type II HAR commonly involves the replacement of the hemiarch or distal anastomosis in zone 1 or 2. Zone 2 arch replacement is of great value in patients with difficult to approach or fragile LSA, which is then either covered or debranched through a cervical incision. There is also an option of perfusing the LSA with a parallel endograft or a branched endograft. Type II HAR is preferred when the diameter of the ascending aorta is near 4 cm to prevent any incidence of RTAD. Like Type I HAR, the stent-graft can be deployed in an antegrade fashion in the same setting. However, staged TEVAR is the favored strategy due to conflicting post-operative management strategies for both stages (28).

### Type III HAR

Type III HAR is an extension of type II HAR with the use of an elephant trunk in the DTA to create a longer PLZ for a second stage TEVAR. Frozen elephant trunk is now frequently considered a variant of type III hybrid repair (29, 30). Type III HAR, primarily intended for disease in the DTA, is the most invasive of all the hybrid approaches, with a 30-day mortality of up to 12% (31). However, type IIIf HAR induces positive remodeling of the dissected aorta by expanding the true lumen and inducing thrombosis of the false lumen. In addition, type IIIf has the advantage of shortening the interval period before the second stage TEVAR, thus decreasing the otherwise significant interval mortality.

### Zone 1 and 2 HAR

Zone I and II hybrid repair involve cervical approaches for supra-aortic vessel transposition or bypass, followed by deployment of stent-graft in zone 1 or 2 depending on the extent of disease in the arch and availability of the landing zone. Zone 1 HAR requires bilateral cervical incision for debranching compared to zone 2 hybrid repair which only needs LCA to the left subclavian artery (LSA) bypass.

### Zone 0 HAR

Zone 0 hybrid repair requires cervical debranching as in zone 1 HAR. Zone 0 HAR is for patients with aortic arch disease that requires coverage of the innominate artery ostium, but with a suitable zone 0 PLZ. Precautions similar to type In HAR needs to be applied to prevent RTAD. Perfusion of the innominate artery and hence to the bypassed grafts will require additional use of a snorkel stent-graft (Zone 0s), or a single branched aortic endograft (Zone 0b) like Nexus Stent Graft System from Endospan (Herzlia, Israel), and Castor branched endograft from MicroPort Medical Co., Ltd. (Shanghai, China).

Zone 0s repair is performed in single stage as incision for cervical debranching also provides direct access to the right common carotid artery (RCCA) for delivery of the parallel stent graft in a retrograde fashion (32).

Ability to use cervical incisions for debranching and thus avoidance of sternotomy makes these hybrid approaches a good option for redo patients. On the other hand, total endovascular arch repair using a multiple branched endograft is not a hybrid repair as it does not involve an open component.

## Outcomes

In a study of 28 patients undergoing type I HAR and eight patients with type II HAR, Bavaria et al. reported in-hospital mortality of 8%, with paraplegia and stroke rates of 5.5 and 8% (33). All the in-hospital mortality was in type I group. Freedom from all-cause mortality at 1, 3, and 5 years was 71, 60, and 48%. Anderson et al., in a study of 87 patients undergoing HAR reported in-hospital mortality of 5.7%, which increased to a 30-day mortality of 14.9% due to out-of-hospital deaths (27). Type In HAR was the only significant risk factor for 30-day mortality. During a separate analysis for type I hybrid group, the composite incidence of RTAD was 6.3%, which increased to 11.1% in type In HAR group. High incidence of RTAD in the type In group translated into increased 30-day mortality in this group compared to type Id patients (29.6 vs. 9.5%). The Kaplan-Meier survival estimate for the entire cohort was 73, 60, and 51% at 1, 3, and 5 years, respectively. Of the 207 supra-aortic vessel bypasses performed in this patient population, 1% were occluded during mean follow-up of 28.5 ± 22.2 months and, interestingly, were clinically silent. Czerny et al., also reported 10.8% incidence of RTAD in patients with type In HAR (34).

A retrospective study of 65 patients undergoing type I and II HAR reported a 42% incidence of late complications, including delayed development of endoleaks, RTAD, stent-graft induced new entry tear (SINE), endograft migration/fracture, and sudden death, with a median time interval of 36.6 months after the initial procedure (35). The incidence of delayed-type 1 endoleak was 14%, primarily proximal (type 1a). In this study, eight patients developed type 1 endoleak and RTAD 6–8 years after the index procedure, thus implying a need for a close long-term monitoring of patients undergoing HAR. Incidence of late sudden death was 6.8% in patients with aortic arch aneurysm and no underlying cardiac or respiratory disease. Authors also recommended reintervention in the event of development of these complications as mortality rate was 100% in patients undergoing conservative management. Importantly. supra-aortic vessel bypasses were not the source of complications or required reintervention and all the arch branches were patent during follow-up. Another study confirmed the mid-term stability of cervical bypasses in a cohort of 23 patients, with no incidence of stenosis or thrombosis during a mean follow-up of 44 months (36). Moulakakis et al., in a meta-analysis of 956 patients undergoing HAR reported a pooled 30-day mortality of 11.9%. Stroke and paraplegia occurred in 7.6 and 3.6% of patients, respectively (37). Endoleak was found in 16.6% of patients with predominance of type I endoleak. Incidence of RTAD was 4.5%. This large meta-analysis showed that HAR can be accomplished even in high risk patients with outcomes comparable to conventional open techniques.

### Hybrid arch repair compared to open aortic arch repair

Benrashid and colleagues in a retrospective study compared the outcomes of hybrid arch repair (101 patients) and open arch repair (47 patients). Even though the hybrid group was significantly older and with a more significant burden of comorbidities, aorta-specific 5-year survival was similar (38). Preventza et al., in a comparative study of 274 patients undergoing traditional open repair, and 45 patients with HAR with zone 0 deployment reported similar survival (hybrid 87.5% vs. traditional 77.2%; *p* = 0.14) at a median follow-up of 4.5 years (39). Operative mortality and incidence of permanent neurologic injury were also similar between the two propensity-matched groups. Interestingly, in this study there was no incidence of RTAD in the hybrid group, even though native ascending aorta was used as a PLZ in almost all the patients. They also reported no permanent spinal cord injury. Authors attributed this to the policy of cerebrospinal fluid (CSF) drainage if the extent of stent-graft in the DTA is more than 15 cm and the low threshold for placing a CSF drain at the onset of early postoperative paraparesis. Another comparative study of an open arch repair and type I HAR in a small group of patients demonstrated similar perioperative mortality rates and stroke (40). Freedom from all-cause mortality at 1, 3, 5, and 7 years was also identical. The results were noteworthy as both the groups were comparable at the baseline. However, this was a retrospective database study in a small cohort of patients. Moreover, the hybrid group experienced significantly higher re-intervention rates (Open repair 14.5% vs. HAR 44.8%, *p* = 0.045).

An attempt to review literature for the Cochrane database to compare HAR with open arch repair in 2021 did not find a single randomized controlled trial or controlled clinical trial to be included in the study and was thus stopped (41). Conventional endograft and recent investigational devices have not yet received FDA approval to treat aortic arch pathologies. Therefore, open arch repair remains the gold standard for treating aortic arch diseases (42).

## Complications

Despite the increasing experience of the operator and refinement of technology, endovascular repairs continue to be plagued by some specific complications like stroke, paraplegia, endoleaks, RTAD, stent induced new entry tears (SINE), stent-graft migration, device malfunction, stent-graft infection, fistulae formation and access-site issues (43). Crowding of the origin of the supra-aortic arteries and curvature of the aortic arch, along with higher blood velocity and increased pulsatility makes hybrid repairs more complex and prone to complications then a TEVAR procedure.

A Consensus Statement From the International Aortic Arch Surgery Study Group (IASSG) in an effort to standardize the clinical end points in the aortic arch surgery argued for the adaptation of a grading system for complications as proposed by Dindo et al. (44, 45). This grading system will facilitate objective assessment and stratification of any adverse event based on its burden and avoid overlapping of results.

### Spinal cord injury

Intercostal, lumbar, hypogastric, and subclavian arteries contribute to the anterior spinal artery, and the intraspinal and paraspinal blood flow networks are interconnected with an extensive network of collaterals (46, 47). In addition, experimental studies have shown the anterior spinal artery's tremendous ability to dilate after the occlusion of segmental arteries (48). Another experimental study has shown the virtue of this compensatory network in a staged repair, with pigs randomized to hybrid aortic repair in a staged fashion experiencing no spinal cord injury (SCI) compared to 50% incidence in pigs treated in a single stage (49).

SCI is a catastrophic complication after endovascular repair the aorta, with a large meta-analysis showing the incidence of 4% after stent-graft deployment in the DTA (50). In the setting of the coverage of segmental arteries with a stent-graft, additional insults like hypotension, reperfusion injury, insufficient collateral network due to pre-existing vascular disease or aortic intervention, and micro-embolization contribute to the SCI. Micro-embolization is now a well-established cause, with a study showing evidence of scattered MRI lesions in 80% of patients with SCI after thoracoabdominal aortic (TAA) repairs (51). Other established risk-factors for the development of SCI are exclusion of LSA, extent of aortic coverage, large bore ileo-femoral sheaths, long procedure with episodes of hypotension, and renal insufficiency.

A large study of 1,251 patients undergoing endovascular aortic repair showed that more than half of the cases of SCI present in a delayed fashion and that nearly half of these patients with delayed SCI had a clear precipitating event. The study also showed the poor 3-month survival of patients with permanent SCI compared to patients with only transient SCI (36 vs. 92%, *p* < 0.01) (52). Delayed SCI is the result of an episode of hypoperfusion of the spinal cord already susceptible to ischemic insult by the endograft's previous coverage of the segmental arteries. Salvage maneuvers to recover the spinal cord function should be focused on increasing the perfusion pressure and maximizing oxygen delivery. Maintaining a higher mean arterial pressure (MAP) after a stent-graft deployment is of paramount importance as has been been shown by a study from the University of Pennsylvania, in which the mean MAP was 74 mm Hg at the onset of delayed SCD after TAA repair, and 94 mm Hg at the time of recovery (53). We also know that patients who experience immediate or early SCI have lesser chance of recovery.

Miyamoto et al., and Dr. Cooley, through their experimental work in the early 1960 s, established the role of drainage of CSF in increasing the blood flow to the spinal cord and thus preventing paraplegia (54, 55). Bavaria group has reported mean CSF pressure of 14 mm Hg at the onset of delayed paraplegia and <10 mm Hg at the time of recovery, with 19% increase in spinal cord perfusion pressure with 4 mm Hg drop of CSF pressure. Authors also reported that 75% of delayed paraplegia event happens in the first 48 h, with a median interval of 21.6 h after the procedure (53). Some groups are more aggressive and advocate target CSF pressure of <5 mm Hg (56). To avoid the complication of cerebral herniation and hemorrhage associated with rapid CSF drainage, the rate of CSF drainage should not exceed 10 ml/h, 25 ml/4 h, and 150 ml/day. To be able to institute rescue maneuvers, it is imperative to keep a close watch on the neurological status for the first 72 h. CSF catheter is maintained for at least 48 h to prevent the onset and aid in the recovery from any episode of SCI. MAPs are maintained between 90 and 100 mm Hg. MAP >120 mm Hg is treated to avoid hypertensive complications.

Measures to prevent “second-hit” and occurrence of SCI should focus on preventing hemodynamic instability with low threshold for cardioversion for an episode of atrial fibrillation, control of any bleeding, aggressive treatment of hypovolemic, cardiogenic, or vasoplegic shock. Body temperature should be controlled to maintain vascular tone.

In the event of SCI, CSF catheter is checked for malfunction, and rapidly inserted at the bedside if not in place already, to maintain CSF pressure <10 mm Hg. MAP target should be increased to 100–115 mm Hg with the help of vasopressors. Some centers put patients in trendelenburg position to help with the blood pressure, and volume status should be optimized with a goal central venous pressure of 10–12 mm Hg. Over correction of volume status can be detrimental by increasing the CSF pressure and thus decreasing perfusion. Hemoglobin level should be maintained to more than 10 gm/dL to optimize oxygen delivery. Mannitol and dexamethasone should be considered to aid in the recovery and limit spinal cord edema. Lidocaine and magnesium sulfate should be administered if there is no recovery in 6–12 h. Paraplegia lasting more than 48 h is likely permanent and warrants palliative care like prophylaxis for deep venous thrombosis, and bladder and bowel management.

Other adjuncts maneuvers include use of intrathoracic papaverine, hyperbaric oxygen therapy, and acetazolamide in an attempt to reduce CSF production (57–59).

### Stroke

Perioperative stroke is another major limitation of HAR with pooled rate of 7.6% in a large meta-analysis (37). Embolization is the leading cause of perioperative stroke. Atherosclerotic plaques in the aortic arch is not uncommon. In the Stroke Prevention: Assessment of Risk in a Community (SPARC) study, more than half of the 581 random samples of Olmsted County, Minnesota, older than 45 years had evidence of arch atheroma on a transesophageal echo, while 7.6% had severe atheroma, defined as at least 4 mm thick, ulcerated or mobile (60). Incidence of severe atheroma was more than 20% in subjects more than 75 years of age. An autopsy study reported a 28% prevalence of ulcerated plaques in the aortic arch in patients dying of cerebrovascular disease compared to only 5% in those dying of some other cause (61). Another autopsy study found an increasing prevalence of severe atheroma in the ascending aorta with age (62).

Trauma and resultant dissection of the arteries and cerebral hypoperfusion during debranching are known causative factors for stroke. Incidence of stroke is most significant with zone 0 endograft deployment attributed to the need for more extensive debranching and endovascular manipulation (63). This also explains the higher occurrence of stroke after HAR than TEVAR, as reported by Freezor et al., in a series of 196 patients (64). In a propensity-matched analysis of 319 patients undergoing open arch repair and HAR, incidence total neurologic event was higher in the HAR group compared to the open repair (17.8 vs. 8.0%; *p* = 0.051) (65).

The complexity of endovascular intervention involving the aortic arch increases the chances of embolization. Limitations of the current delivery system can also result in “snowplowing” of the debris from the arterial wall. Identifying high-risk patients by carefully studying the cerebral vessel during operative workup can help surgeons formulate a plan to minimize or avoid intervention in a vulnerable region. Exchange of wires and catheters should be kept to a minimum.Use of fluoroscopic guidance while advancing wires, and performance of advanced wire manipulation like snaring distal to the arch can help reduce the incidence of stroke. Unnecessary manipulation of cerebral vessels should be avoided during the debranching procedure to avoid embolization and stroke.

Hypoperfusion during debranching of the supra-aortic arteries can also result in a stroke. Clamp-test of the cerebral vessels for 2 min to confirm the absence of any EEG change before debranching adds extra protection to the procedure. In the event of EEG changes, debranching should be done on cardiopulmonary bypass under moderate hypothermia and higher perfusion pressure.

### Endoleak

Endoleaks are classified as per the modified EVAR reporting standards (66) and are the major cause of failure and aortic reintervention after a hybrid procedure. Endoleaks, with a reported incidence between 15 and 25% after HAR, are the major cause of technical failure, reintervention, large mortality, and even disseminated intravascular coagulopathy, and aortic rupture (67, 68). A study stratifying the incidence of endoleaks based on PLZ of the stent-grafts found the lowest rate of endoleaks with zone 0 deployment and the highest with zone 1 deployment (7.1 vs. 33.3%) (69). A meta-analysis confirmed a higher incidence of endoleak with zone 1 deployment compared to zone 0 deployment (15.48 vs. 3.97%; *p* = 0.0050), with a higher reintervention rate in zone 1 group, during an average follow-up of 21.6 months (25.81 vs. 12.0%; *p* = 0.0321) (70). Primary type I and III endoleaks were responsible for the higher rate in patients with zone 1 deployment and have been attributed to the short length of the landing zone and angulation of the aortic arch.Close apposition of the stent-graft to the aortic wall and coiling of the proximal LSA prevents type II endoleak. Type I endoleak is a significant cause of late failure and reintervention and is related to the complexity of landing the graft in the arch or ascending aorta. Angulation of the ascending aorta and arch with the higher pulsatile flow in the region is responsible for “bird-breaking,” of the stent-graft resulting in type I endoleak and stent migration. Success and adaptation of the total endovascular aortic arch repair with zone 0 PLZ is also primarily limited by type I endoleak, especially type I gutter-leak associated with parallel stent-grafts.

### Retrograde type A dissection

A large meta-analysis of 8,969 patients reported pooled incidence of 2.5% for RTAD after endovascular repair (71). The study also showed RTAD to be associated with very high mortality rate (37.1%). Williams et al., in a series of 309 patients, where rate of RTAD was 1.9%, identified zone 0 PLZ and ascending aorta diameter >4 cm as risk-factors for the development of RTAD (72). Clamp injury to the native aorta, compliance mismatch between the rigid aortic graft and native tissue, greater pulsatility in the proximal aorta, exposed barbs of the stent-grafts have all been postulated as the cause of RTAD after HAR. In a retrospective study of 66 patients undergoing type I HAR, rate of RTAD after type In HAR was found to be 10.6% (34). Duke University group also reported the RTAD incidence of 11.1% after type In HAR. As discussed before, RTAD was postulated as a factor for out of hospital death and high 30-day mortality (29.6%) in type I HAR group (27). Close long-term follow-up is recommended after HAR due to the known risk of delayed RTAD. Connective tissue disorder is also associated with a higher incidence of RTAD (8.3%) (73). Direction of stent-graft deployment (antegrade vs. retrograde) has not been found to be associated with RTAD or endoleaks.

## Controversies

### Overstenting of left subclavian artery

Maintenance of flow in the left subclavian artery, while relevant to cerebral perfusion, is also critical in patients with previous coronary artery bypass with patent mammary artery. Access to the LSA for the debranching can be difficult through a sternotomy in obese patients and with large aneurysms. LSA can be fragile and calcified making manipulation extremely challenging, especially if the artery is aneurysmal or dissected. In patients with deep seated LSA, debranching can be accomplished by cervical bypasses or transposition. A transthoracic aorta to infraclavicular axillary bypass through the left first or second intercostal space is also an option. It is important to anticipate this problem especially if planning to deploy the stent-graft in an antegrade fashion in the setting of debranching.

A study of 606 patients from a prospective database reported significantly higher rates of neurologic complications with LSA coverage when compared to patients undergoing prophylactic revascularization (8 vs. 0%, *p* = 0.049) (74). A systematic review of 2,591 patients undergoing TEVAR with LSA coverage documented a significantly lower perioperative stroke rate in patients with LSA revascularization than in patients without revascularization (RR 0.61; 95% CI 0.45–0.82; *I*^2^ = 20%) (75). In a study where 121 patients had over-stenting of LSA without revascularization, the incidence of left upper-extremity malperfusion was 9.9% (76). Revascularization of the LSA is not benign, as has been documented in a study where the rate of short-term complications after a carotid-subclavian bypass was 29%, most of which were attributed to phrenic nerve palsy (25%). Other reported complications were recurrent laryngeal and axillary nerve injury, lymph leak and bleeding (77).

A retrospective study of a prospectively maintained database, where only 22% of patients underwent a carotid-subclavian bypass for over-stenting of the LSA, showed that selective revascularization of LSA does not increase the risk of neurologic complications (78). Rates of stroke and paraplegia in patients with and without revascularization were similar (stroke: 3.1 vs. 3.5%; *p* > 0.99, paraplegia: 0.1 vs. 0%; *p*−0.22). Authors recommended revascularization of LSA if right SCA is absent, with dominant left vertebral artery, prior coronary bypass grafting with patent LITA, and patent left axillary-femoral bypass. LSA should also be revascularized in patients at higher risk of SCI, like prior abdominal aortic repair, extensive DTA coverage with endograft, and if the left hand is dominant. Patients with mega-aorta or extensive DeBakey type 1 dissection, who will likely require additional aortic procedures should also be considered for LSA revascularization. Current guidelines recommend at least selective pre-operative revascularization of LSA in elective cases, direct debranching or trans-thoracic bypass for emergent cases, and close post-operative monitoring if not revascularized for need for urgent reintervention (79, 80). Several studies have confirmed the excellent long-term patency after both carotid-subclavian bypass and sublclavian-carotid transposition (81, 82). During transposition, it is crucial to divide the LSA before the origin of the vertebral artery, and proximal LSA should be ligated during the carotid-subclavian bypass to prevent a type II endoleak. If access to the proximal LSA is difficult, then coils or plug should be placed in the LSA during staged endograft deployment. Dueppers et al., compared endovascular vs. open debranching of the LSA in a cohort of 48 patients. Although, technical success was lower in the the endovascular group, both the groups showed no significant difference in thirty-day mortality, rates of neurologic complications, and freedom from all-cause mortality (83). Early type I endoleak was more prevalent in the endovascular group, and most of them were gutter-related.

### Patients with connective tissue disorder

As per the Genetically triggered thoracic aortic aneurysms and cardiovascular conditions (GenTAC) registry, a multi-institutional database of confirmed or suspected genetically triggered thoracic aortic pathology, connective tissue disorders like Marfan syndrome, Loeys-Dietz syndrome, vascular Ehlers-Danlos syndrome are more likely to suffer complications after endovascular repair and with high mortality (84). Self-expanding stent-graft in a fragile and diseased aorta in patients with connective tissue disorder has been known to cause increased incidence of RTAD, stent migration, and SINE) (85). Stent-graft is generally avoid in connective tissue disorder patients except in the presence of multiple comorbidities, and in redo patient with availability of Dacron graft as a landing zone. HAR can also be offered to a patient presenting with malperfusion in the setting of aortic dissection as salvage therapy with the hope of a definitive repair in the future. Close life-long follow-up is warranted in such patients for the need of reintervention.

### Our experience

As mentioned in this review, open surgical repair has been the traditional approach for patients with aneurysmal degeneration of the aortic arch and descending thoracic aorta. Often staged, this approach, however, is fraught with considerable morbidity, including risks of stroke (1–20%), spinal cord injury (1–8%), and death (0–20%). To mitigate these risks, our institution has recently adopted a staged HAR approach as an alternative treatment strategy for these complex aortic pathologies.

Since transitioning to a staged HAR approach, between November 2019 to March 2022, our institution has performed 35 consecutive stage I HAR, with 34 patients receiving subsequent staged TEVAR (97%) and one death (2.9%) precluding second stage intervention. Of the 35 patients, median age was 60 years old, 17 patients had a previous history of cardiac surgery (15 for type A aortic dissection), there was a preponderance of male patients (80%), and the majority had a history of hypertension (89%). Thirty-two patients underwent repair for aortic dissection (92%); of this cohort, 17 were labeled as acute, 14 were chronic and 1 subacute. This is in contrast with our previous institutional experience, where indications for repair were primarily degenerative aneurysms of the aortic arch.

During initial presentation, 27 patients received a type II HAR (our current preferred method), 6 patients received type III HAR (prior to our adoption of type II HAR) and 2 patients underwent a type I HAR. Our preference is to perform a carotid-subclavian bypass with the aim to reduce the operative and ischemic times, however, as most patients are operated on an urgent basis for acute dissection, it is not always feasible. As such, 25 patients did not undergo LSA debranching or revascularization at the time of stage I HAR. Among them, 15 received a carotid-subclavian bypass during a subsequent procedure 3–7 days after stage I operation. One patient died prior to TEVAR, and 9 patients underwent stage II TEVAR without LSA revascularization.

In our experience, median interval time from stage I HAR to second stage TEVAR was 13 days, with 18 completed within the index hospitalization. This is in contrast to the traditional approach, where patients historically experienced prolonged intervals of up to 6 months between stages, with interval mortality seen in up to 27% of patients (86, 87).

Overall 30-day mortality for the entire population was 3.3% (1 mortality). Following stage I HAR, there was no incidence of stroke or myocardial infarction. There was an 11% incidence of new-onset renal failure requiring dialysis (*n* = 4), 40% of patients required mechanical ventilatory support for more than 48 h (*n* = 14) and 8% of patients returned to the operating room for re-exploration (*n* = 8). Following stage II repair, there was no incidence of 30-day or 90-day mortality, stroke, or paraplegia. There was a 3.3% incidence for both new-onset renal failure requiring dialysis (*n* = 1), as well as re-exploration (*n* = 1). Three patients (9%) have required reintervention following discharge after stage II repair; one required Zone 5 TEVAR for SINE, one required Zone 3 TEVAR for mycotic pseudoaneurysm, and one required redo arch replacement for a kinked graft.

While our experience is limited to short-term outcomes, hopefully future long-term results will help determine the durability of HAR in patients with complex aortic pathologies. However, we believe that the low overall morbidity and mortality in the acute setting that we demonstrated, argues that HAR can be safely extended to patients with isolated aneurysmal disease and suggest that HAR be considered for all patients with dissecting and aneurysmal diseases of the aortic arch.

## Author contributions

SS and SP: research and draft of manuscript. RA and PV: research, draft of manuscript, and review. All authors contributed to the article and approved the submitted version.

## Conflict of interest

The authors declare that the research was conducted in the absence of any commercial or financial relationships that could be construed as a potential conflict of interest.

## Publisher's note

All claims expressed in this article are solely those of the authors and do not necessarily represent those of their affiliated organizations, or those of the publisher, the editors and the reviewers. Any product that may be evaluated in this article, or claim that may be made by its manufacturer, is not guaranteed or endorsed by the publisher.

## Abbreviations

CPB: cardiopulmonary bypass
CSF: cerebrospinal fluid
DTA: descending thoracic aneurysm
HAR: hybrid aortic arch repair
HCA: hypothermic circulatory arrest
IASSG: international aortic arch surgery study group
LCA: left common carotid artery
LSA: left subclavian artery
MAP: mean arterial pressure
PLZ: proximal landing zone
RCCA: right common carotid artery
RTAD: retrograde type a aortic dissection
SCI: spinal cord injury
SPARC, stroke prevention: assessment of risk in a community study
SINE: stent induced new entry tears
TEVAR: thoracic endovascular aortic repair.
